# Improved diagnostic performance of CASPAR criteria with integration of ultrasound

**DOI:** 10.3389/fimmu.2022.935132

**Published:** 2022-10-10

**Authors:** Yan Geng, Zhibo Song, Xiaohui Zhang, Xuerong Deng, Yu Wang, Zhuoli Zhang

**Affiliations:** Department of Rheumatology and Clinical Immunology, Peking University First Hospital, Beijing, China

**Keywords:** psoriatic arthritis, diagnostic utility, CASPAR criteria, ultrasound, integration

## Abstract

**Background:**

The difficulty in determining synovitis, tenosynovitis, or enthesitis by physical examination (PE) has limited the diagnostic capability of CASPAR for psoriatic arthritis (PsA). Therefore, we aimed to evaluate the diagnostic utility of CASPAR with the integration of ultrasound (US).

**Methods:**

Patients with a hint of PsA were enrolled. Besides routine PE for tender or swollen joints, enthesitis, and dactylitis, US was performed to evaluate peripheral joints, entheses, and tendons. The additional value of the US to the CASPAR criteria was analyzed.

**Results:**

A total of 326 consecutive patients with 164 PsA and 162 non-PsA were enrolled. A total of 162 non-PsA patients consisted of 58 cases of psoriasis (PsO), 27 osteoarthritis with PsO/family history of PsO, five fibromyalgia with PsO, 69 sero-negative rheumatoid arthritis, and three undifferentiated arthritis. Significantly higher frequencies of tenosynovitis and enthesitis on US and new bone formation on X-rays were found in PsA vs. non-PsA patients (59.1% *vs*. 13.0%; 63.4% *vs*. 14.2%; 62.2% *vs*. 8.0%, *p <*0.01 for all). Logistic regression analysis showed that dactylitis (OR = 12.0, *p <*0.01), family history of PsO/PsA (OR = 3.1, *p <*0.05), nail involvement (OR = 3.5, *p =* 0.01), new bone formation on X-ray (OR = 14.8, *p <*0.01), tenosynovitis on US (OR = 21.3, *p <*0.01), and enthesitis on US (OR = 21.7, *p <*0.01) were independent risk factors for PsA. By combining US tenosynovitis and/or enthesitis, the diagnostic utility of CASPAR criteria was improved, with superior specificity (91.4% *vs*. 84.0%) and similar sensitivity (95.7% *vs*. 94.5%). Replacing X-ray by US or adding US, the CASPAR criteria showed comparable sensitivity and specificity for PsA diagnosis. The diagnostic accuracy was 89.3% for CASPAR criteria based on PE, 93.6% for CASPAR added with US, and 93.3% for CASPAR with US replacing X-ray.

**Conclusion:**

The diagnostic utility of the CASPAR was improved by integrating tenosynovitis and/or enthesitis when using US. US provides additional value for PsA recognition.

## Introduction

Psoriatic arthritis (PsA) is a chronic inflammatory disease, manifesting as peripheral arthritis, enthesitis, dactylitis, or spondylitis besides skin and nail psoriasis (1). Peripheral joints and entheses are the most commonly involved domains. Moreover, inflammatory articular disease is the prerequisite for CASPAR (ClASsification criteria for Psoriatic Arthritis), the most widely used criteria in the diagnosis of PsA (2). Nevertheless, it is often difficult to determine the cause of synovitis, tenosynovitis, enthesitis, or dactylitis by physical examination (PE) alone.

In recent years, ultrasound (US) has been recognized as a feasible, reliable, and non-radiative tool, and it has been widely used in assessing inflammatory arthritis. Some previous studies have also demonstrated that subclinical synovitis and enthesitis identified by US are common in PsA and even in some psoriasis (PsO) patients. On the other hand, overestimation of inflammatory articular disease also happens in practice due to osteoarthritis or fibromyalgia (3). Therefore, the European League Against Rheumatism (EULAR) recommended detecting arthritis, tenosynovitis, and enthesitis in peripheral spondyloarthritis by application of US instead of clinical examination only to improve the diagnostic accuracy (4).

Although the CASPAR criteria have been validated, CASPAR based on PE is not the “gold standard.” The final diagnosis of PsA is usually made by experienced rheumatologists after considering all the available evidence. In this study, we tried to explore the contribution of US on the basis of CASPAR criteria to the diagnosis of PsA.

## Materials and methods

### Study population and participants

We conducted a cross-sectional study on patients with suspected PsA at Peking University First Hospital. The patients were enrolled from June 2019 to May 2021. In detail, patients with the following clinical features were included: 1. Presence of PsO/family history of PsO plus at least one of the following: (1) presence of tender and/or swollen joint on physical examination; (2) tender entheses on physical examination; (3) swollen digits with/without tender on physical examination and 2. The absence of PsO/family history of PsO, being seronegative, but physical examination revealed tender and/or swollen joints, or tender entheses, or swollen digits with/without tender. Those suspected PsA patients with axial involvement were excluded. The exclusion criteria were as follows: 1. use of disease-modifying antirheumatic drugs within 3 months before enrollment; and 2. steroid therapy (oral and intra-articular) or non-steroidal anti-inflammatory drugs within 2 weeks before enrollment. The research protocol was approved by the Peking University First Hospital Institutional Review Board for clinical research and consent forms were obtained from all participants in compliance with the Helsinki Declaration.

### Clinical and laboratory assessment

The demographics, including age, sex, family history of PsO, and body mass index (BMI), were recorded. The duration of arthralgia and/or enthesis pain was recorded. The following variables were collected and further calculated: swollen joint counts (SJC), tender joint counts (TJC) of 46 joints [bilateral elbows, wrists, metacarpophalangeal joints, proximal interphalangeal joints, distal interphalangeal joints, knee, ankle, and metatarsophalangeal joints], global assessment of patients (0–10), and global assessment of physicians (0–10). Tenderness of 16 entheses (bilateral proximal patellar tendon, distal patellar tendon, quadriceps tendon, Achilles’ tendon, plantar aponeurosis, common extensor tendon, common flexor tendon, and triceps tendon) and dactylitis on 20 digits (bilateral hands and feet) were examined. Psoriasis was scored using the psoriasis area and severity index (PASI). Nail involvement was recorded. The following laboratory tests were recorded: erythrocyte sedimentation rate, C-reactive protein, rheumatoid factor (RF), and anti-cyclic citrullinated peptide (anti-CCP).

### Ultrasound and X-ray assessment

**Figure 1:**
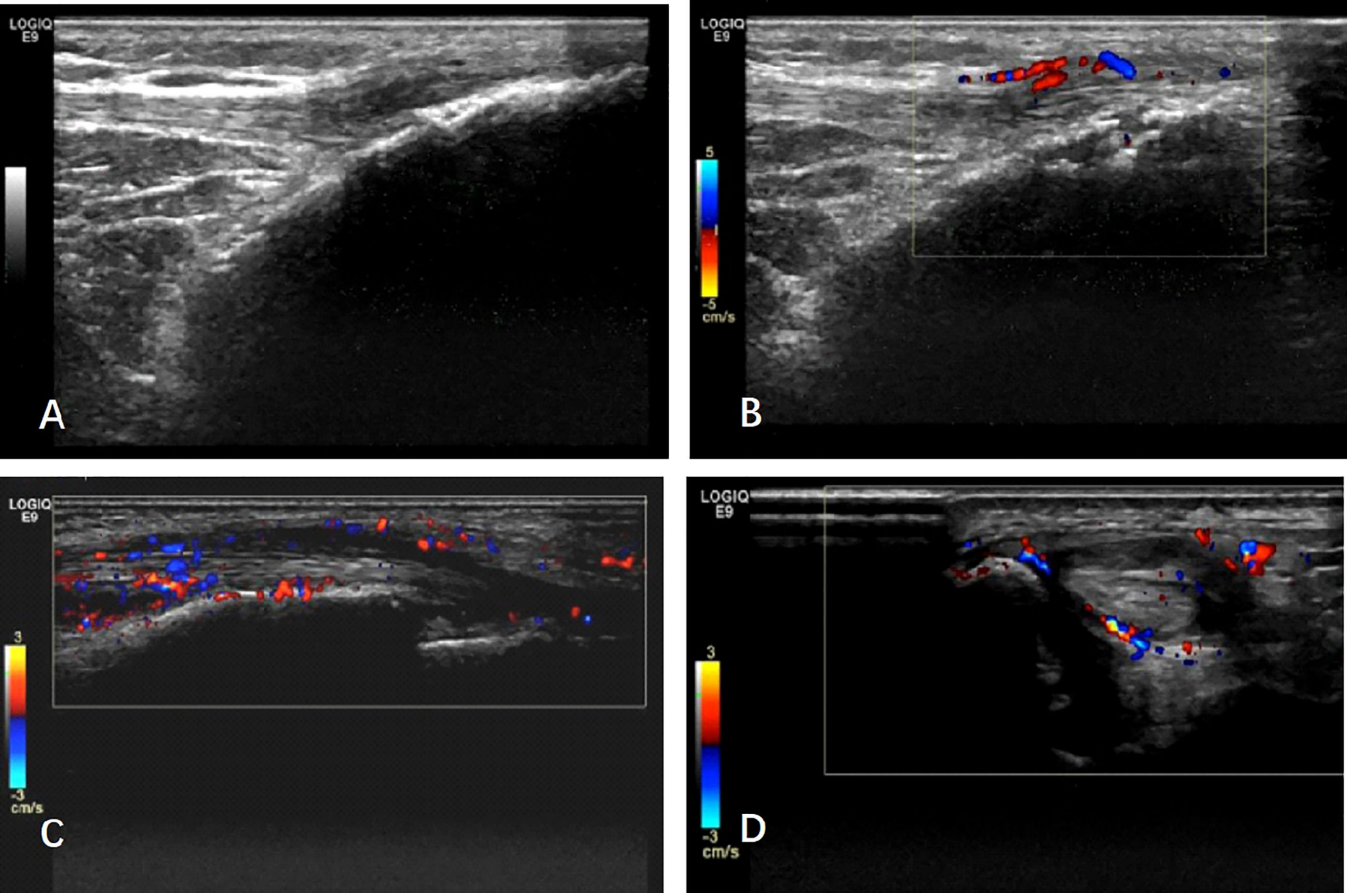
The typical ultrasound image of psoriatic arthritis: **(A, B)** Enthesitis: **(A)** longitudinal scan of patellar tendon at its distal insertion into the anterior tibial tuberosity. Note the presence of hypoechoic areas, entheseal thickening together with an enthesophyte. **(B)** B-mode signs of enthesitis are detectable. The image shows evidence of power Doppler signal at the entheseal area, together with erosion. **(C, D)** Tenosynovitis: longitudinal and transverse scan of anterior tibialis tendon. Note abnormal anechoic and hypoechoic tendon sheath widening with intense power Doppler signals.

US examination was performed by a rheumatologist (XRD) who was the ultrasound trainer endorsed by EULAR with over 10 years of experience in maneuvering US and was blinded to all clinical and laboratory findings. A total of 46 joints (same to TJC and SJC), 16 entheses (same to tenderness count), and 36 tendons (flexor and extensor tendon of digit, compartments of extensor tendons of wrist, posterior tibialis tendon, and anterior tibialis tendon) were scanned. The US protocol included transverse and longitudinal scans of the joints and enthesis. Each scan took at least 20 min, and the representative images were archived. The GE LOGIQ E9 US machine with linear ML 15–6 MHz or small-footprint linear array 18–8 MHz transducers was used in our study. The gray-scale and Doppler settings were as below: wall filter low, pulse repetition frequency (PRF) 1.0 kHz, and gain was adjusted to just below the level at which Doppler artifacts appeared beneath bone. The severity of synovitis was measured and graded using the 2001 Szkudlarek semi-quantitative method (5). Gray Scale (GS) and Power Doppler (PD) synovitis scores in each joint were respectively graded on a scale of 0–3. GS ≥2 or PD ≥1 for a joint was defined as synovitis (6). Enthesitis was defined as hypoechoic and/or thickened insertion of the tendon close to the bone (within 2 mm from the bony cortex), which exhibits Doppler signal if active, and which may show erosions and enthesophytes/calcifications as a sign of structural damage (7) (**Figures 1A, B**).

Tenosynovitis was defined as abnormal anechoic and/or hypoechoic tendon sheath widening with or without PD signals (**Figures 1C, D**). Erosion was defined as intra-articular discontinuity of the bone surface observed in two perpendicular planes (8). The effects of synovitis, enthesitis, tendon involvement, and erosion on the US were analyzed in a dichotomous way.

A posterior–anterior X-ray of the hands and feet was taken for all the patients. New bone formation was evaluated by a radiologist who was blinded to all clinical data (9).

### Diagnostic criteria

For analyzing the diagnostic accuracy of US features and the additional value of US to CASPAR, the clinical diagnosis of PsA made by experienced rheumatologists was taken as the standard. All controversial cases were reviewed by a panel of three experts (ZZ, YG, and ZS) who were blinded to the US findings.

### Statistical analysis

All statistical analyses were performed using Statistical Package of Social Science (SPSS Inc., Chicago, IL, USA) software v. 22.0. A T-test or Mann–Whitney U-test was used for measurement data, and χ^2^ test was used for categorical data in the comparative analysis between groups. The analysis based on the receiver operating characteristic (ROC) curve was performed to determine the optimal cut-off value for the best combination of sensitivity and specificity. Logistic regression was used to predict the independent risk factors for the diagnosis of PsA. P-values <0.05 were considered as being significant. P-values <0.01 were considered as highly significant.

## Results

### Comparisons of demographics and clinical features between PsA and non-PsA groups

Three hundred and twenty-six patients were enrolled in the study. Clinically, 164 were diagnosed as PsA. A total of 162 non-PsA consisted of 58 PsO, 27 osteoarthritis with PsO/family history of PsO, five fibromyalgia with concomitant PsO, 69 sero-negative rheumatoid arthritis, and three undifferentiated arthritis.

The demographics and clinical characteristics of patients with PsA and non-PsA are shown in **Table 1**. Their average age was 48.3 years. More patients reported a family history of PsO/PsA in the PsA group than in the non-PsA group (43.9% *vs*. 25.9%, *p <*0.01). The PASI score was higher in the PsA group than in the non-PsA group (6.6 ± 9.6 *vs*. 3.0 ± 8.0, *p <*0.01). More patients had clinical enthesitis, dactylitis, and nail involvement in the PsA group than in the non-PsA group (20.1% *vs*. 9.9%, p <0.05; 35.4% *vs*. 2.5%, p <0.01; 58.3% *vs*. 24.7%, *p <*0.01, respectively). The presence of RF or anti-CCP was very low, with no statistical significance between the two groups.

**Table 1:** Comparisons of the demographics and clinical features between PsA and non-PsA groups.

	PsA group(n = 164)	Non-PsA group (n = 162)	P
**Demographic characteristics**			
Female, n (%)	65 (39.6%)	105 (64.8%)	<0.01
Age (years)	46.2 ± 13.5	48.3 ± 16.0	0.102
BMI (kg/m^2^)	25.1 ± 4.1	25.4 ± 6.3	0.947
Family history of PsO/PsA, n (%)	72 (43.9%)	42 (25.9%)	<0.01
**Clinical characteristics**			
Joint symptom duration (years)	4.7 ± 6.3	3.9 ± 5.4	0.113
Tender joint count, n	5.8 ± 6.2	3.7 ± 5.4	<0.01
Swollen joint count, n	4.0 ± 4.5	2.0 ± 4.1	<0.01
Enthesitis, n (%)	33 (20.1%)	16 (9.9%)	0.015
Dactylitis, n (%)	58 (35.4%)	4 (2.5%)	<0.01
PGA (0–10), mm	3.6 ± 2.2	3.0 ± 2.8	0.067
PhGA (0–10), mm	3.5 ± 2.1	2.8 ± 2.6	0.017
Nail involvement, n (%)	95 (58.3%)	40 (24.7%)	<0.01
PASI score	6.6 ± 9.6	3.0 ± 8.0	<0.01
**Laboratory parameters**			
ESR (mm/h)	21.4 ± 24.0	21.2 ± 21.6	0.121
CRP (mg/L)	12.6 ± 21.2	11.4 ± 17.5	0.365
RF positive, n (%)	2 (1.2%)	5 (3.7%)	0.275
Anti-CCP positive, n (%)	2 (1.2%)	2 (1.2%)	1.000
**US characteristics**			
Synovitis, n (%)	98 (59.8%)	75 (46.3%)	0.015
Tenosynovitis, n (%)	97 (59.1%)	21 (13.0%)	<0.01
Enthesitis, n (%)	104 (63.4%)	23 (14.2%)	<0.01
Erosion, n (%)	84 (51.2%)	38 (23.5%)	<0.01
Osteophyte, n (%)	89 (54.3%)	88 (54.3%)	0.992
**X-ray characteristics**			
New bone formation	102 (62.2%)	13 (8.0%)	<0.01

The measurement data were presented as mean ± SD unless it was indicated specifically. BMI, body mass index; PsA, psoriatic arthritis; PsO, psoriasis; PGA, patient global assessment; PhGA, physician global assessment; PASI, Psoriasis Area and Severity Index; ESR, erythrocyte sedimentation rate; CRP, C-reactive protein; RF, rheumatoid factor; anti-CCP, anti-cyclic citrullinated peptide; US, ultrasound.

Among US characteristics, synovitis was found in 59.8% of PsA patients and 46.3% of non-PsA patients (*p <*0.05). The presence of tenosynovitis, enthesitis and bone erosion was significantly more in the PsA group than in the non-PsA group (59.1% *vs*. 13.0%; 63.4% *vs*. 14.2%; 51.2% *vs*. 23.5%; *p <*0.01 for all). Compared to the non-PsA group, a significantly higher proportion of patients in the PsA group had new bone formation on X-ray (62.2% *vs*. 8.0%, *p <*0.01).

### Independent risk factors for predicting the diagnosis of PsA

Age, sex, family history of PsO/PsA, PASI score, nail involvement, dactylitis, new bone formation on X-ray, and various US features were included in the multivariate analysis to identify the possible predicting factors. We found that dactylitis (OR = 12.0, 95% CI 2.7–53.5, *p <*0.01), nail involvement (OR = 3.5, 95% CI 1.4–9.3, *p =* 0.01), family history of PsO/PsA (OR = 3.1, 95% CI 1.2–8.4, *p <*0.05), new bone formation on X -ray (OR = 14.8, 95% CI 5.3–41.4, *p <*0.01) and tenosynovitis on US (OR = 21.3, 95% CI 6.8–66.9, *p <*0.01), enthesitis on US (OR = 21.7, 95% CI 7.7–61.4, *p <*0.01) were independent risk factors for predicting the diagnosis of PsA (**Table 2**).

**Table 2:** The risk factors for predicting PsA by multivariate logistic regression analysis.

Parameters	OR (95% CI)	P
Dactylitis	12.0 (2.7,53.5)	0.001
Family history of PsO/PsA	3.1 (1.2–8.4)	0.022
Nail involvement	3.5 (1.4–9.3)	0.010
PASI score	1.0 (0.9–1.1)	0.343
New bone formation on X-ray	14.8 (5.3–41.4)	<0.01
Synovitis on US	0.9 (0.4–2.5)	0.943
Tenosynovitis on US	21.3 (6.8–66.9)	<0.01
Enthesitis on US	21.7 (7.7–61.4)	<0.01
Erosion on US	2.7 (0.9–7.5)	0.055

PsA, psoriatic arthritis; PsO, psoriasis; PASI, Psoriasis Area and Severity Index; US, ultrasound.

### Diagnostic values of US features for PsA

Among US characteristics, tenosynovitis and enthesitis were significantly more commonly observed in the PsA group and were identified as independent risk factors for predicting PsA. In this study, tenosynovitis in the US showed 59.1% sensitivity and 87.0% specificity for the diagnosis of PsA. Enthesitis in the US showed 63.4% sensitivity and 85.8% specificity in diagnosing PsA. In contrast, the CASPAR criteria based on physical examination had a high sensitivity of 94.5% but a relatively low specificity of 84.0%.

### The added value of US to the CASPAR criteria

Since US features, including tenosynovitis and enthesitis, were risk factors for predicting PsA, we subsequently added US tenosynovitis and/or enthesitis to the CASPAR scoring system. The presence of US tenosynovitis and/or enthesitis was given the same weight as nail involvement (1 point), and the cut-off total score of ≥4 was used to classify a patient as having PsA.

**Figure 2:**
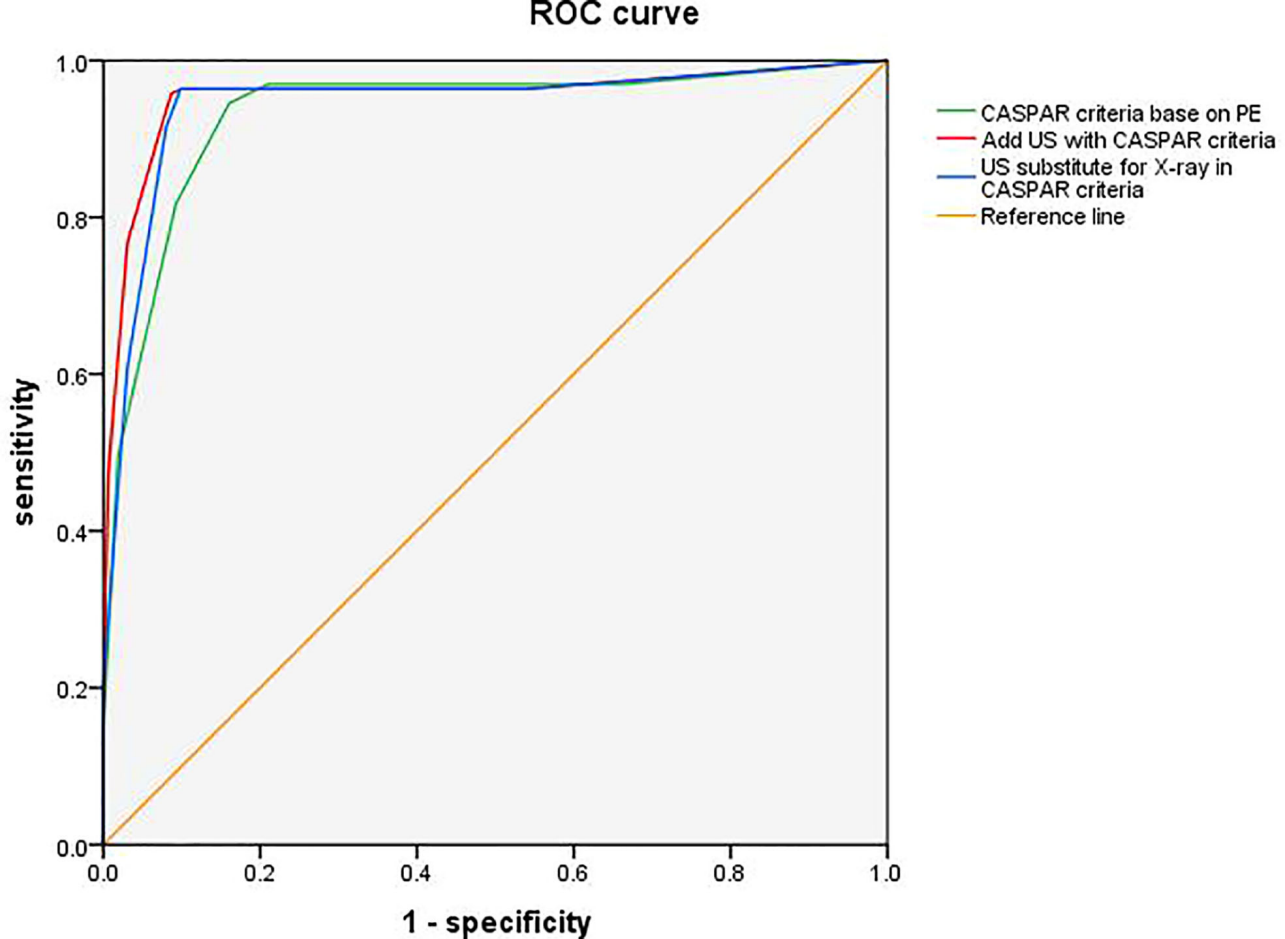
Receiver operating characteristic (ROC) curves for adding US or substituting X-ray by US in CASPAR. ROC curve illustrates the diagnosis performance of CASPAR with US added, CASPAR with replacing X-ray by US, and CASPAR based on physical examination only. The corresponding areas under the ROC curves (AUC) were 0.954 (95% CI 0.928, 0.979; p <0.01), 0.944 (95% CI 0.916, 0.972; p <0.01), and 0.933 (95% CI 0.903, 0.962; p <0.01). CASPAR, ClASsification criteria for Psoriatic Arthritis; US, ultrasound.

Compared to the original CASPAR criteria based on PE, the modified CASPAR criteria integrated with US findings showed better performance, with improved specificity (91.4% *vs*. 84.0%) and similar sensitivity (95.7% *vs*. 94.5%) (**Figure 2**). Diagnostic accuracy was improved from 89.3% to 93.6% (*p = 0.052)*, with better positive predictive value (91.8% *vs*. 85.6%) and comparable negative predictive value (95.5% *vs.* 93.8%).

An X-ray is an invasive procedure and is incapable of disclosing inflammation. It is always ideal if an X-ray can be substituted by US. Therefore, we tried replacing X-rays in the CASPAR criteria with US. Unexpectedly, we found both the sensitivity and specificity were noninferior to CASPAR criteria with combined US (96.3% *vs*. 95.7% and 90.1% *vs*. 91.4%) (**Figure 2**). The diagnostic accuracy was also comparable (93.3% *vs*. 93.6%, *p = 0.250*), with a positive predictive value of 90.8% and a negative predictive value of 96.1%.

The ROC curve illustrated the diagnostic performance of two modified CASPAR criteria added with US, and substituting X-ray by US, as well as the original CASPAR criteria. The corresponding areas under the ROC curves (AUC) were 0.954 (95% CI 0.928, 0.979; <0.01), 0.944 (95% CI 0.916, 0.972; *p* <0.01), and 0.933 (95% CI 0.903, 0.962; *p* <0.01).

## Discussion

CASPAR is most widely used in the diagnosis of PsA in practice. Its utility has been assessed by a series of studies, showing relatively high specificity and sensitivity. The better performance of CASPAR than the Moll and Wright criteria as well as the European Spondyloarthropathy Study Group criteria has also been demonstrated (10).

Early identification of PsA is crucial for a better long-term outcome. A previous study showed that even a 6-month delay of PsA diagnosis in a rheumatology clinic resulted in worse outcomes, including more peripheral joint erosion and functional impairment (11). Thus, close attention to the joint symptoms and a comprehensive physical examination are needed to identify the inflammatory articular disease. Nevertheless, it is always difficult to precisely identify synovitis and enthesitis by swelling and/or tenderness of the joint or entheseal *via* physical examination (12). Moreover, PsO patients with concomitant osteoarthritis or fibromyalgia often easily satisfy the CASPAR criteria, leading to over-diagnosis of PsA. This may explain the result of good sensitivity but poor specificity of CASPAR in our study.

US has been validated as a useful tool in evaluating joint, tendon, and entheseal lesions in PsA (13–15). But few studies evaluated the overall value of US in addition to clinical findings in the diagnosis of PsA (16). In this study, 326 patients with suspected PsA were included and detected for various pathological changes, including inflammatory and chronic lesions on US. We found higher frequencies of synovitis, tenosynovitis, enthesitis, and bone erosion in PsA patients compared with the non-PsA group. Among these lesions, tenosynovitis and enthesitis were identified as independent risk factors for PsA. Further analysis revealed significantly improved specificity and fair sensitivity of CASPAR for the diagnosis of PsA when US tenosynovitis (87.0% and 59.1%) or US enthesitis (85.8% and 63.4%) were incorporated. In line with our study, Zabotti et al. reported that the presence of at least one extra-synovial feature in hands on US was significantly associated with early PsA, with specificity of 88.1% and sensitivity of 68.0% (17). Qiu et al. found that joint synovitis, bone erosions, tenosynovitis, and enthesitis on US were more frequently observed in PsA patients than in non-PsA patients. Tendon sheath synovial thickening showed the highest sensitivity (78.5%), while PD signal and bone erosion of enthesis showed high specificities (94.6% and 96.9% respectively) for PsA (18). A systemic review of 20 studies indicated that US tenosynovitis was highly specific (95%–100%) but entheseal lesions showed considerable variation in specificity (33%–99%) (19). There were several possible reasons to explain these discrepancies, for instance, different lesions and sites assessed, diverse enrolled patients, and study designs. In the study, we used a similar US protocol to the UPSTREAM study, but we additionally scanned four joints (bilateral elbow and ankle) and four entheses (bilateral common flexor tendon and triceps tendon). The US protocol in revealing typical changes in PsA has been validated by a few studies (20, 21).

The recognition of PsA by either the US alone or CASPAR based on physical examination alone was unsatisfactory. But when US was used with CASPAR, the specificity was increased from 84.0% to 91.4% while keeping the sensitivity for PsA diagnosis. Although based on limited evidence (22), US assessment integrated with clinical assessment has been proposed to improve the early identification of PsA. Our study confirmed the diagnostic value of the US evaluation for CASPAR. An X-ray is an invasive procedure and is incapable of disclosing inflammation. Although new bone formation on an X-ray is a characteristic feature of PsA, standing for long-term structural damage secondary to inflammation, and it is therefore unhelpful for early diagnosis. US, in contrast, is a sensitive, reliable, and non-radiative tool. Therefore, it would be beneficial if the X-ray could be substituted with US. In our study, when we modified the CASPAR by substituting the X-ray with US, both the sensitivity and specificity were as good as the modified CASPAR with US added. The area under the ROC curve was also similar between *the* modified CASPAR criteria. For the same diagnostic performance, the use of a radiation-free imaging modality is preferable. Therefore, US should be recommended as a substitute for X-rays in CASPAR.

The advantages of this study are the comprehensive evaluation of US in addition to clinical assessment and the large number of patients enrolled in a single center study. We are aware of some limitations. First, the PsA and non-PsA patients in our study were not sex-matched, but this bias was corrected by the multivariate statistical analysis. Second, US features were not evaluated quantitively and did not distinguish between inflammatory or structural damage components. The US scan protocol is complicated and time-consuming. Future studies on establishing a simplified US score system that encompasses both joint and extra-articular structures are warranted. Third, using only one US examiner for all patients in our study reduced the inter-observer bias by its maximum but could not guarantee intra-observer reliability. Fourth, the conclusions from this single-center study still require external validation.

## Conclusion

The modified CASPAR (the integration of US) improves the diagnosis utility. Moreover, X-rays can be substituted with US, which is a valuable tool in assisting the diagnosis of PsA in clinical practice.

## Data availability statement

The raw data supporting the conclusions of this article will be made available by the authors, without undue reservation.

## Ethics statement

This study was reviewed and approved by the Ethics Committee of Peking University First Hospital. The patients/participants provided their written informed consent to participate in this study.

## Author contributions

ZZ conceived of the study and critically revised the manuscript. YG had full access to all the data collection, analysis, interpretation, and drafted the manuscript. ZS, XZ, XD, and YW contributed to the data collection. All authors contributed to the article and approved the submitted version.

## Funding

This work was supported by the Interdisciplinary Clinical Research Project of the Peking University First Hospital (2021CR30), the Youth Clinical Research Project of the Peking University First Hospital (2019CR28), and the National Natural Science Foundation of China (grant nos. 81901646, 81801611, 81971524 and 81771740).

## Acknowledgments

The authors would like to thank all the patients and rheumatology nurses who contributed to our study. Patients or the public were not involved in the design, conduct, reporting, or dissemination plans of our research.

## Conflict of interest

The authors declare that the research was conducted in the absence of any commercial or financial relationships that could be construed as a potential conflict of interest.

## Publisher’s note

All claims expressed in this article are solely those of the authors and do not necessarily represent those of their affiliated organizations, or those of the publisher, the editors and the reviewers. Any product that may be evaluated in this article, or claim that may be made by its manufacturer, is not guaranteed or endorsed by the publisher.
